# Vitamin C and COVID-19

**DOI:** 10.3389/fmed.2020.559811

**Published:** 2021-01-18

**Authors:** Harri Hemilä, Angelique M. E. de Man

**Affiliations:** ^1^Department of Public Health, University of Helsinki, Helsinki, Finland; ^2^Department of Intensive Care Medicine, Amsterdam University Medical Centers, Amsterdam, Netherlands

**Keywords:** artificial respiration, ascorbic acid, coronavirus, critical care, pneumonia, respiratory distress syndrome, respiratory tract infections

## Abstract

In numerous animal studies, vitamin C has prevented and alleviated viral and bacterial infections. In a few dozen placebo-controlled trials with humans, vitamin C has shortened infections caused by respiratory viruses, which indicates that the vitamin can also influence viral infections in humans. In critically ill patients, plasma vitamin C levels are commonly very low. Gram doses of vitamin C are needed to increase the plasma vitamin C levels of critically ill patients to the levels of ordinary healthy people. A meta-analysis of 12 trials with 1,766 patients calculated that vitamin C reduced the length of ICU stay on average by 8%. Another meta-analysis found that vitamin C shortened the duration of mechanical ventilation in ICU patients. Two randomized placebo-controlled trials found statistically significant reduction in the mortality of sepsis patients. The effects of vitamin C on acute respiratory distress syndrome (ARDS) frequently complicating COVID-19 pneumonia should be considered. Vitamin C is a safe and inexpensive essential nutrient.

## Introduction

About 100 animal studies have shown that vitamin C can prevent and alleviate many kinds of viral and bacterial infections (1, 2). Because of the great diversity in the infectious agents in those studies, it is evident that the effect of vitamin C is not restricted to any specific virus or bacterium. Furthermore, in the early literature, pneumonia was reported to be common in patients suffering from vitamin C deficiency (3, 4), which also indicates that the vitamin can have clinically relevant effects in the protection against infections in humans. Vitamin C may improve the immune response to viral infections through the stimulation of the proliferation and function of T-lymphocytes and NK-lymphocytes, and the production of interferon (2, 5, 6).

## Evidence Indicating That Vitamin C Might Influence COVID-19

Several of the numerous animal studies on vitamin C and infections (1, 2) are relevant when considering the potential role of the vitamin against the new SARS-CoV-2 coronavirus. Vitamin C increased the resistance of chick embryo tracheal organ cultures to infection caused by an avian coronavirus (7), and protected broiler chicks against an avian coronavirus (8). In addition, in septic mice with acute respiratory distress syndrome (ARDS), vitamin C administration downregulated proinflammatory genes, enhanced epithelial barrier function, and improved alveolar fluid clearance (9, 10). In addition, the deficiency of vitamin C increased lung pathology caused by influenza A in mice (11).

A number of controlled vitamin C trials in humans are also important when considering the new coronavirus. A few dozen placebo-controlled trials with humans showed that regularly administered ≥1 g/day vitamin C shortened infections caused by respiratory viruses in adults by 8%, and in children by 18% (12). Respiratory viruses form a heterogeneous group and their distribution varies over time and location. Therefore, types of viruses have varied between the trials and it is unlikely that the benefit of vitamin C is explained by effects on just a certain respiratory virus or virus group. Because the effect of vitamin C on the diverse group of respiratory viruses seems non-specific, it seems plausible that vitamin C may also have effects on the new coronavirus.

In the placebo-controlled trials on the common cold (12), the magnitude of effect of regularly administered vitamin C has not been very large and does not justify regular vitamin C supplementation in normal situations. However, the new coronavirus causes an illness that is much more severe than ordinary respiratory virus infections and frequently causes pneumonia complicated by ARDS. Therefore, even moderate benefits of an 8–18% decrease in the duration of respiratory virus infections would justify consideration of vitamin C supplementation. Moreover, in the early literature, pneumonia was described as a common complication of frank vitamin C deficiency, scurvy, and two small controlled trials indicated that vitamin C might have therapeutic benefits against pneumonia (3, 4).

The particular concern with COVID-19, the disease caused by the novel coronavirus, is that ICU treatment is needed for a rather high proportion of patients. There is much evidence that critically ill patients have reduced plasma levels of vitamin C, which is explained by the increased depletion of the vitamin in their body so that one third of ICU patients may have as low vitamin C levels as vitamin C deficient patients (13, 14). In particular, a recent survey found that out of 18 COVID-19 patients, 17 had undetectable vitamin C levels and one patient had a very low level (15). Another recent study also reported low vitamin C plasma levels in COVID-19 patients, and non-survivors had half the plasma level of survivors (16). Although 0.1 g/day of vitamin C can maintain ordinary plasma levels in healthy persons (17), critically ill patients need much higher doses (2–3 g/day) to increase the plasma vitamin C levels to the ordinary range (13, 18). It would therefore seem reasonable to screen plasma vitamin C levels in ICU patients and administer vitamin C to those with low levels. Unfortunately, vitamin C assay with HPLC is quite expensive and therefore not usually available in daily practice, and the cheaper tests are less accurate.

A meta-analysis of 12 controlled trials with 1,766 patients found that vitamin C had shortened ICU stay on average by 8% (13). Another meta-analysis of eight trials found that vitamin C shortened the duration of mechanical ventilation in patients who needed the longest ventilation (19). Furthermore, Zabet et al. (20) reported that vitamin C reduced mortality in 28 sepsis patients by 78% (*P* = 0.01; based on 2/14 vs. 9/14) and Fowler et al. (21) reported that vitamin C reduced mortality in 167 patients with sepsis and ARDS by 35% (*P* = 0.01; based on 25/84 vs. 38/83). A reanalysis of the latter trial showed that during the 4-day vitamin C administration, mortality was decreased in the vitamin C group with RR = 0.19 (95% CI 0.06–0.55) (22). During the 4-day intervention, the number needed to treat was 5.5 (95% CI 3.5–12.5), which means that one death was prevented in five to six patients by vitamin C (22).

## Discussion

Although there is as yet no direct evidence indicating that vitamin C is beneficial specifically against COVID-19, the reported benefits of vitamin C in the ICU context suggest that it could be considered for patients. Based on the dose vs. plasma level analyses, it is unlikely that a healthy person would benefit from daily vitamin C doses over 0.5 g/day (17). However, for patients suffering from a respiratory virus infection, 6–8 g/day of oral vitamin C was significantly more effective than 3–4 g/day (1). In the recent studies with patients with sepsis (20) and with sepsis and ARDS (21–23), the doses of intravenous vitamin C were 7–16 g/day for 3–4 days.

Vitamin C is an essential, inexpensive nutrient. Due to the severe clinical course of COVID-19 pneumonia, even moderate benefits may be worthwhile. However, the excellent safety profile of vitamin C and the necessity of ICU treatment for a high proportion of COVID-19 patients may justify consideration of clinical application of vitamin C, even before the results of large clinical trials are available (24). Vitamin C has been proposed for COVID patients also by other authors (25–27).

## Author Contributions

HH and AM participated in the revision of the manuscript.

## Conflict of Interest

The authors declare that the research was conducted in the absence of any commercial or financial relationships that could be construed as a potential conflict of interest.
